# Adoptive transfer of allergen-expressing B cells prevents IgE-mediated allergy

**DOI:** 10.3389/fimmu.2023.1286638

**Published:** 2023-11-23

**Authors:** Lisa Prickler, Ulrike Baranyi, Konstantinos Mengrelis, Anna Marianne Weijler, Verena Kainz, Bernhard Kratzer, Romy Steiner, Jasmin Mucha, Elisa Rudoph, Nina Pilat, Barbara Bohle, Herbert Strobl, Winfried Franz Pickl, Rudolf Valenta, Birgit Linhart, Thomas Wekerle

**Affiliations:** ^1^ Division of Transplantation, Department of General Surgery, Medical University of Vienna, Vienna, Austria; ^2^ Cardiac Surgery Research Laboratory, Department of Cardiac Surgery, Medical University of Vienna, Vienna, Austria; ^3^ Institute of Immunology, Center for Pathophysiology, Infectiology and Immunology, Medical University of Vienna, Vienna, Austria; ^4^ Department of Pathophysiology and Allergy Research, Center for Pathophysiology, Infectiology and Immunology, Medical University of Vienna, Vienna, Austria; ^5^ Division of Immunology and Pathophysiology, Otto Loewi Research Center for Vascular Biology, Immunology and Inflammation, Medical University of Graz, Graz, Austria; ^6^ Karl Landsteiner University of Health Sciences, Krems an der Donau, Austria; ^7^ Institute of Immunology Federal Medical-Biological Agency (FMBA) of Russia, National Research Center (NRC), Moscow, Russia; ^8^ Laboratory of Immunopathology, Department of Clinical Immunology and Allergy, Sechenov First Moscow State Medical University, Moscow, Russia

**Keywords:** allergy, prophylaxis, tolerance, cell therapy, chimerism

## Abstract

**Introduction:**

Prophylactic strategies to prevent the development of allergies by establishing tolerance remain an unmet medical need. We previously reported that the transfer of autologous hematopoietic stem cells (HSC) expressing the major timothy grass pollen allergen, Phl p 5, on their cell surface induced allergen-specific tolerance in mice. In this study, we investigated the ability of allergen-expressing immune cells (dendritic cells, CD4^+^ T cells, CD8^+^ T cells, and CD19^+^ B cells) to induce allergen-specific tolerance in naive mice and identified CD19^+^ B cells as promising candidates for allergen-specific cell therapy.

**Methods:**

For this purpose, CD19^+^ B cells were isolated from Phl p 5-transgenic BALB/c mice and transferred to naive BALB/c mice, pre-treated with a short course of rapamycin and an anti-CD40L antibody. Subsequently, the mice were subcutaneously sensitized three times at 4-week intervals to Phl p 5 and Bet v 1 as an unrelated control allergen. Allergen-expressing cells were followed in the blood to monitor molecular chimerism, and sera were analyzed for Phl p 5- and Bet v 1-specific IgE and IgG_1_ levels by RBL assay and ELISA, respectively. *In vivo* allergen-induced lung inflammation was measured by whole-body plethysmography, and mast cell degranulation was determined by skin testing.

**Results:**

The transfer of purified Phl p 5-expressing CD19^+^ B cells to naive BALB/c mice induced B cell chimerism for up to three months and prevented the development of Phl p 5-specific IgE and IgG_1_ antibody responses for a follow-up period of 26 weeks. Since Bet v 1 but not Phl p 5-specific antibodies were detected, the induction of tolerance was specific for Phl p 5. Whole-body plethysmography revealed preserved lung function in CD19^+^ B cell-treated mice in contrast to sensitized mice, and there was no Phl p 5-induced mast cell degranulation in treated mice.

**Discussion:**

Thus, we demonstrated that the transfer of Phl p 5-expressing CD19^+^ B cells induces allergen-specific tolerance in a mouse model of grass pollen allergy. This approach could be further translated into a prophylactic regimen for the prevention of IgE-mediated allergy in humans.

## 1 Introduction

IgE-mediated allergic diseases are an increasing health problem in industrialized countries (1). Allergic patients develop an aberrant T and B cell response to otherwise innocuous antigens, i.e., allergens, resulting in allergen-specific IgE production and subsequent activation of effector cells by allergen/IgE immune complexes (2). The investigation of IgE responses to allergens in birth cohort studies has revealed that initial allergic sensitization occurs early in life (3). In grass pollen-allergic children, sensitization was shown to begin long before the onset of symptoms, and the major timothy grass pollen allergen, Phl p 5, was among the most frequently recognized allergens in the preclinical phase (4). Moreover, it has been suggested that the sensitization pattern acquired in childhood remains stable in adults (5).

There are few causative therapy options available to treat an established allergy, like allergen-specific immunotherapy (6) and anti-IgE treatment, which share the disadvantage of having limited efficacy and being expensive (7). However, effective prophylactic approaches to prevent the onset of allergies are still an urgent medical need (8). Studies in mice and humans have shown that allergen-specific IgG antibodies may protect infants against allergic sensitization (9, 10). Accordingly, it has been suggested to induce protective allergen-specific IgG responses by vaccinating children early in life or even pregnant mothers so as to transfer these antibodies to their offspring (11–13). Moreover, the induction of oral tolerance to food allergens by the early introduction of allergenic food into the diet or by administration of tolerogenic allergen-derived peptides has been proposed, but clinical studies have led to controversial results (11, 14–16).

Cell therapy is a strategy with the potential to prevent or treat immunological diseases (17, 18). The establishment of hematopoietic chimerism is a type of cell therapy that is effective in inducing antigen-specific tolerance in transplantation and autoimmunity. It refers to the transfer of allogenic cells or autologous cells that express the disease-causing antigen(s) into appropriately conditioned recipients (19–22). Transplantation of donor hematopoietic stem cells (HSC) has been investigated as a strategy for tolerance induction in transplantation and has successfully led to tolerance to kidney transplantation in pilot clinical trials (23). This approach has been extended to other immunological diseases, like thalassemia and aplastic anemia (24–27).

We have applied the chimerism approach to IgE-mediated allergy and developed a prophylactic protocol based on the transfer of allergen-expressing bone marrow (BM) cells. Autologous BM cells were retrovirally transduced *in vitro* to express clinically relevant pollen allergens, Phl p 5 or Bet v 1, and induced robust and long-lasting allergen-specific tolerance upon adoptive transfer (28, 29). This approach showed two peculiarities: permanence and robustness, as it was demonstrated that tolerance persisted for the entire length of the 40-week follow-up period. Specific tolerance was proven at the T cell, B cell, and effector cell levels, all relevant for a Type 1 allergic immune response. By long-term monitoring of chimerism levels in the peripheral blood, it was shown that microchimerism (i.e., very low levels of chimerism) is sufficient to maintain IgE-antibody tolerance (30). Subsequently, a transgenic mouse model expressing the Phl p 5 allergen on the surface of all body cells, along with cytoplasmic expression of green fluorescence protein (GFP), was generated as a cell donor to conduct adoptive transfer and tolerance induction studies. In the next step, an irradiation-free adoptive cell transfer protocol was established that induced long-term tolerance to the specific allergen in mice exclusively conditioned by a short course of co-stimulation blockage and mTOR inhibition (31).

Bone marrow (BM) as a cell source for adoptive cell transfers requires invasive measures for cell harvesting (either BM aspiration or drug-induced HSC mobilization). Moreover, it is undetermined which cell subsets need to express the antigen of interest to induce tolerance by cellular chimerism. Therefore, we herein investigated if allergen-expressing peripheral blood resident immune cell subsets instead of HSCs could induce IgE-specific tolerance upon adoptive transfer.

## 2 Materials and methods

### 2.1 Mouse strains

Female BALB/c mice (6-12 weeks old) were purchased from Charles River Laboratories (Sulzfeld, Germany). The Phl p 5 IRES GFP BALB/c transgenic mouse, which ubiquitously expresses the major timothy grass pollen allergen, Phl p 5, on the cell surface and eGFP intracellularly on a BALB/c background, has been described previously (31) and will be referred to as the Phl p 5 tg mouse. Phl p 5 tg mice were bred at the animal facility of the Medical University of Vienna. The transgene was detected by PCR as described previously (31). Co-expression of intracellular GFP with membrane-bound Phl p 5 was detected in blood, spleen, and lymph nodes by flow cytometric analysis, and GFP was therefore used as a reasonable marker for Phl p 5 expression (**Supplementary Figure 1**). Mice were kept at the animal facility of the Medical University of Vienna under protected conditions. All experiments were approved by the ethics votum of the Austrian Federal Ministry of Education, Science, and Research (Permission number GZ: GZ 2021-0.276.441 and BMWFW-66.009/0028-WF/V/3b/2015) and were performed in accordance with national and international guidelines for the care of laboratory animals.

### 2.2 Development of a mouse model for allergen-specific tolerance induction

**Figure 1:**
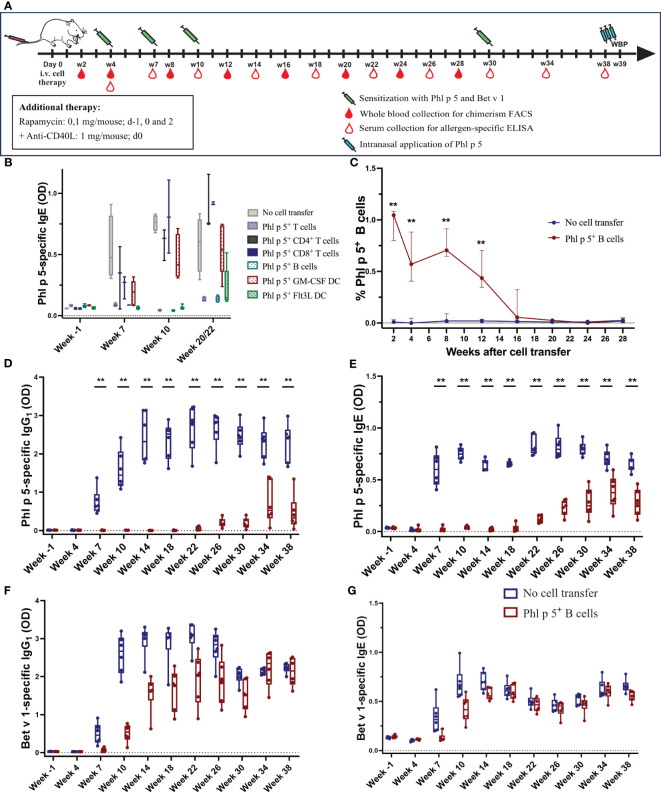
Phl p 5^+^ B cell therapy with rapamycin and an anti-CD40L antibody induces B cell chimerism and leads to long-term tolerance **(A)** Mouse experimental scheme of cell therapy in naïve recipient mice. BALB/c recipient mice (6-8 weeks) received pre-treatment with anti-CD40L (1 mg on day 0) and a short course of rapamycin (0.1 mg on days -1, 0, and 2) prior to cell transfer from a Phl p 5-transgenic mouse. Mice were sensitized to Phl p 5 and Bet v 1 at weeks 4, 7, 10, and 30. Serum and peripheral blood were collected at the indicated time points. Recombinant Phl p 5 was administered intranasally on days -3, -2, and -1 before WBP. **(B)** The tolerogenic potential of different cell subsets was tested by measuring Phl p 5-specific IgE levels (Y-axis) in serum by ELISA for the no cell transfer group (n=5), Phl p 5^+^ T cells (n=5), Ph l p 5^+^ CD4^+^ T cells (n=3), Phl p 5^+^ CD8^+^ T cells (n=3), Phl p 5^+^ B cells (n=5), Phl p 5^+^ GM-CSF DC (n=5), and Phl p 5+ Flt3L (n=6). B cells were selected as a promising candidate for all subsequent cell therapies. Data from three independent experiments are shown. **(C)** Percentages of Phl p 5^+^ B cells (Y-axis) in peripheral blood were analyzed by flow cytometric analyses in recipient mouse groups (Phl p 5^+^ B cells, n=6 and no cell transfer, n=6) at multiple time points (X-axis). The medians and interquartile range of percentages of Phl p 5^+^ cells are shown on the Y-axis. **(D, E)** Phl p 5-specific and **(F, G)** Bet v 1-specific IgG_1_ and IgE levels in sera from mice treated with Phl p 5^+^ B cells or no cells were measured by ELISA, and OD levels (Y-axis) are shown at indicated time points (X-axis). (Data from one experiment) Results are presented as box plots, and significant P values are shown. (** p<0.01).

Immune cells were obtained from the Phl p 5 tg mouse as follows: T cells were isolated by positive selection with the CD4 (L3T4) and CD8a (Ly-2) T cell isolation kits (Miltenyi Biotec, Bergisch Gladbach, Germany) from the spleen and lymph nodes of Phl p 5^+^ transgenic mouse donors, and CD19^+^ B cells were isolated by negative selection with the Pan B cell isolation kit (Miltenyi Biotec, Bergisch Gladbach, Germany) according to the manufacturer’s instructions and transferred into RPMI media (Merch, Darmstadt, Germany). For granulocyte macrophage colony-stimulating factor (GM-CSF) and FMS-like tyrosine kinase 3 ligands (Flt3L) stimulated dentritic cells (DCs), BM cells were isolated from Phl p 5^+^ transgenic mice, red blood cells were lysed, and BM cells were cultured in RMPI media (Biochrom, Berlin, Germany) supplemented with 10% fetal calf serum (FCS), 100 U/ml penicillin, 100 µg/ml streptomycin, 2 mM L-glutamine, 25 mM Hepes, and 50 µM ß-mercaptoethanol, and with 100 ng/ml rmGM-CSF (Thermo Fisher Scientific, MA, USA) or rmFlt3L (Thermo Fisher Scientific, MA, USA), respectively. Media were changed on days 1, 3, and 5. On day 9, loosely adherent cells were recovered and used as immature GM-CSF- or Flt3L-stimulated DCs and further purified by CD11c positive selection (Miltenyi Biotec, Bergisch Gladbach, Germany). Purity was analyzed by flow cytometry analyses (BD FACS Canto II). BALB/c recipient mice were pre-treated with co-stimulatory blockade anti-CD40L intraperitoneally (i.p.) (MR1; 1mg, d0, BioXcell, NH, USA) and a short course of rapamycin i.p. (0.1mg/mouse, days -1/0/2, LC laboratories, MA, USA) (**Figure 1A**). Mice received 1 x 10^7^ isolated cells of the different immune cell populations (CD19^+^ B cells, CD4^+^ T cells, CD8^+^ T cells, a 1:1 mix of CD4^+^/CD8^+^ T cells, GM-CSF-stimulated DCs, or Flt3L-stimulated DCs) intravenously (i.v.) in the tail vein on d0. Groups were immunized subcutaneously (s.c.) with 5 x 10^6^ Phl p 5 splenocytes and 5 µg rBet v 1 adsorbed to aluminum hydroxide (Alu-Gel-S; Serva, Ingelheim, Germany) at weeks 4, 7, 10, and 30 after cell transfer, as described (31). Blood samples were collected repeatedly up to 38 weeks after cell transfer as indicated in the mouse experimental scheme in **Figure 1A**. Whole blood was used for flow cytometry measurements. Sera were stored at -20°C until analysis.

### 2.3 Detection of allergen-specific IgE and IgG_1_ by ELISA

Allergen-specific IgE and IgG_1_ antibody levels were measured in pre-immune and immune sera of mice as described in (32). Plates were coated with either recombinant Phl p 5 (rPhl p 5) (5 µg/ml) or rBet v 1 (5 µg/ml). Sera were diluted 1/20 for IgE and 1/500 for IgG_1,_ and bound antibodies were detected with monoclonal rat anti-mouse IgE (clone A35-72) and IgG_1_ (clone A85-1) antibodies (BD Bioscience, San Diego, CA, USA) diluted 1/1000 and a goat anti-rat antiserum coupled to horseradish peroxidase (HRP) (BD Biosciences, San Diego, CA, USA) diluted 1/2000, respectively. As substrate for HRP, 2,2′-azino-bis(3-ethylbenzothiazoline-6-sulfonic acid) (ABTS) was used. Extinctions (optical density, 450 to 405 nm) were determined with an ELISA reader (Tecan Infinite F50, Tecan Trading AG, Switzerland).

### 2.4 Flow cytometric analyses

To detect Phl p 5^+^/GFP-expressing CD19^+^ B cells, flow cytometric analyses were performed. The percentage of Phl p 5^+^ cells was calculated by subtracting the number of positive events observed in control staining from those quadrants containing Phl p 5^+^ and Phl p 5^-^ cells expressing a CD19^+^ marker and by dividing the net percentage of Phl p 5^+^ cells by the total net percentage of Phl p 5^+^ plus Phl p 5^-^ CD19^+^ B cells as described (33). Phl p 5-specific polyclonal antibodies were purified from rabbit serum raised against rPhl p 5 on a HiTrap Protein G HP antibody purification column (Merck, Darmstadt, Germany) according to the manufacturer’s instructions. Polyclonal anti-Phl p 5 IgG was used as the primary antibody and developed by counterstaining with PE donkey anti-rabbit IgG. 7AAD (Viability Staining Solution, BioLegend, San Diego, USA) was used to exclude dead cells. CD19-APC-Cy7 (clone: 6D5) and CD11b-PE (clone: M1/70) were purchased from BioLegend (San Diego, USA). CD4-PE-Cy7 (clone: GK1.5), CD45.2-eFluor500 (clone: 104), and CD3- eFluor450 (clone: 17A2) were purchased from Thermo Fisher Scientific (MA, USA). CD8-APC (clone: 53-6-7) and CD45.2-PerCP-Cy5.5 (clone: 104) were purchased from BD Biosciences (San Diego, CA, USA). Flow cytometric analyses were performed using a BD FACS Canto II (BD Biosciences, San Diego, CA, USA), and data were analyzed using FlowJo software version 10.7.2.

### 2.5 Rat basophil leukemia cell degranulation assay

Rat basophil leukemia (RBL) cell mediator release was performed as previously described (28). RBL-2H3 cells were cultured in 96-well tissue culture plates (4 x10^4^ cells/well) (Greiner Bio-One, Stuttgart, Germany) at 37°C with 5% CO_2_ for 24 h. Cells were loaded with 1:40 diluted mouse sera and incubated at 37°C in 5% CO_2_ for 2 h. The cell layer was washed twice with Tyrode’s buffer to remove unbound antibodies. RBL cells were stimulated with 0.01 µg/well rPhl p 5 at 37°C for 30 min. Supernatants were incubated with the substrate 80 µM 4-methylumbelliferyl-N-acetyl-b-D-glucosamide (Merck, Darmstadt, Germany) in citrate buffer at 37°C for 1 h. The reaction was stopped by adding 100 µL of glycine buffer, and the fluorescence was measured at λex:360/λem:465 nm using a fluorescence microplate reader (Tecan Spark, Tecan Trading AG, Switzerland). The results are expressed as a percentage of the total ß- hexosaminidase released after the addition of 10% Triton X- 100 (Merck, Darmstadt, Germany).

### 2.6 Allergens/*In vivo* antibodies

Monoclonal antibodies, anti-CTLA4 (clone 9H10) (1 mg/mouse on d21, 0.5 mg/mouse on d23, d25, and d27), and anti-PD-1 (clone J43) (1 mg/mouse on d21, 0.5 mg/mouse on d23, d25, and d27), were purchased from BioXcell (West Lebanon, NH, USA).

The sequence encoding the timothy grass pollen allergen, Phl p 5a (Genebank Accession No. CAA52753), was cloned into the expression plasmid pET17b (Novagen, Merck, Darmstadt, Germany) using the NdeI and EcoRI restriction sites. The recombinant protein was expressed in *E. coli* BL21 (DE3) cells (Agilent, CA, USA) along with an N-terminal 8x histidine tag and purified by affinity chromatography on a Ni-NTA Superflow Column (Qiagen, Venlo, The Netherlands) according to manufacturer´s instructions. The histidine tag was removed by TEV protease cleavage. Endotoxins were removed using a Pierce High-Capacity Endotoxin Removal Spin Column (Thermo Fisher Scientific, MA, USA) according to the manufacturer´s instructions. Post-removal endotoxin levels were measured on an Endosafe Portable Test System (Charles River, Wilmington, MA) (0.15 EU/mg Phl p 5). Protein purity was analyzed by SDS-PAGE, and protein concentration was determined using a Micro BCA kit (Thermo Fisher Scientific, MA, USA).

Recombinant Bet v 1.0101 (termed rBet v 1) was produced and characterized as previously described (34).

### 2.7 Airway hyperresponsiveness

Whole-body plethysmography (WBP) was performed as previously described (35). Mice received an intranasal application of rPhl p 5 (10 µg per dose in 40 µl PBS) on 3 consecutive days, days -3, -2 and -1, prior to whole-body plethysmography (Buxco Research Systems, Wilmington, NC, USA). Mice were challenged with increasing doses of methacholine (6.25, 12.5, 25, and 50 mg/ml) (Merck, Darmstadt, Germany) and PBS as baseline, and Penh (enhanced pause) was recorded.

### 2.8 Type I cutaneous hypersensitivity reaction

The abdominal skin of the mice was shaved one day before skin testing. Mice were injected i.v. with 100 µl of 0.5% Evans blue (Merck, Darmstadt, Germany) and subsequently injected intradermally with 30 µl of rPhl p 5 and rBet v 1 (0.5 µg/ml each, diluted in PBS) (36). As a negative control, PBS was injected intradermally, while the mast cell-degranulating compound 48/80 (20 µg/ml, Merck, Darmstadt, Germany) was injected as a positive control. Twenty minutes after injection, the mice were sacrificed, and skin reactions to the allergens were evaluated on the inverted skin.

### 2.9 Statistical analyses

The statistical analyses were performed using GraphPad Prism 5.0 (GraphPad Software, Inc., CA, USA). P-values below 0.05 were considered to denote statistical significance. The reported P-values are the results of two-sided Mann-Whitney U tests. Results are presented as box plots or dot plots, respectively. Box plots represent 50% of the values within the boxes, with median values indicated. Whiskers represent minimum and maximum values.

## 3 Results

### 3.1 Phl p 5-expressing immune cells may differ in their ability to induce tolerance

We investigated whether mature immune cells expressing the major timothy grass pollen allergen Phl p 5 on the cell surface could induce allergen-specific tolerance in recipient mice, as previously shown for allergen-expressing BM cells (31). For this purpose, different immune cells were purified from the spleen and lymph nodes of transgenic Phl p 5-expressing donor mice (31) and used in a prophylactic cell therapy approach to prevent sensitization to Phl p 5. According to the experimental treatment scheme shown in **Figure 1A**, groups of mice (**Supplementary Table 1**) received a mix of CD4^+^/CD8^+^ T cells, CD4^+^ T cells, CD8^+^ T cells, or CD19^+^ B cells, together with a short treatment of rapamycin and anti-CD40L. In addition, two populations of bone marrow-derived dendritic cells (DC) were expanded *in vitro* in the presence of either granulocyte macrophage colony-stimulating factor (GM-CSF) or FMS-like tyrosine kinase 3 ligands (Flt3L). As a negative control, one group did not receive any cell transfers. All mice were sensitized to Phl p 5 and the control allergen, Bet v 1, at weeks 4, 7, and 10 after cell transfer, and Phl p 5-specific IgE levels were determined in serum collected at different time points, as indicated (**Figure 1A**).

A profound reduction in Phl p 5-specific IgE responses was found in mice that received either Phl p 5^+^ B cells, the combination of CD4^+^/CD8^+^ T cells administered in a 1:1 ratio, or Flt3L-stimulated DC. However, the latter group showed an increase in Phl p 5-specific IgE levels at weeks 20/22. In mice treated with CD4^+^ T cells, CD8^+^ T cells, or GM-CSF-stimulated DC, Phl p 5-specific IgE levels developed as early as week 7 and increased after each sensitization (**Figure 1B**). Thus, CD19^+^ B cells are among the most promising cell populations for tolerance induction. Considering the practical applicability of the approach, Phl p 5^+^ CD19^+^ B cells were selected for further evaluation as prophylactic cell therapy.

### 3.2 Definition of the conditioning treatment for the transfer of Phl p 5^+^ B cells

Phl p 5^+^ B cells were isolated from the spleens and lymph nodes, respectively, of Phl p 5^+^ mice by negative selection with magnetic beads, typically resulting in a purity of >95% (**Supplementary Figure 2A**). We confirmed the co-expression of cytoplasmic GFP (green) and Phl p 5 (yellow) on the surface of isolated B cells by fluorescence microscopy (**Supplementary Figure 2B**).

In the first set of experiments, the optimum conditioning regimen was determined. Phl p 5^+^ B cells (10x10^6^ cells/mouse) were transferred into BALB/c recipients, which were pre-conditioned with either rapamycin + anti-CD40L or rapamycin + CTLA4Ig, as both treatment regimens showed promising results in previous cell therapy protocols (37, 38). All mice were sensitized three times to Phl p 5 and Bet v 1 at weeks 4, 7, and 10. Mice treated with rapamycin together with CTLA4Ig failed to develop Phl p 5-specific tolerance, as Phl p 5-specific IgE and IgG_1_ responses were detected as early as three weeks after the first sensitization and increased after the second and third sensitizations. In contrast, mice treated with rapamycin and anti-CD40L did not develop any Phl p 5-specific IgG_1_ and IgE but showed preserved antibody responses to Bet v 1 (**Supplementary Figure 3**). Thus, rapamycin, together with anti-CD40L, allowed cell therapy with Phl p 5 B cells to induce allergen-specific tolerance.

### 3.3 Transplantation of Phl p 5^+^ B cells induces B cell chimerism and long-term tolerance

Mice were treated according to the experimental scheme shown in **Figure 1A**. The percentage of Phl p 5^+^ B cells in peripheral blood was determined by flow cytometric analysis upon i.v. injection of Phl p 5^+^ B cells under the rapamycin/anti-CD40L conditioning regimen described above. Phl p 5^+^ B cells were detectable for 16 weeks post-transplant. Chimerism peaked at 1.05% of peripheral blood B cells two weeks after cell transfer and dropped over time to less than 0.5% at week 12 and could barely be detected at week 16 (**Figure 1C**).

As we assumed that tolerance induction occurred soon after cell transfer, we analyzed the presence of Phl p 5^+^ B cells in the spleen, lymph nodes, bone marrow, and thymus on days 2, 7, and 21 by flow cytometric analysis. The highest numbers of Phl p 5^+^ B cells were detected in the spleen, followed by lymph nodes and bone marrow, until day 21. Interestingly, Phl p 5^+^ B cells were also detected in the thymus, but only until day 7 (**Supplementary Figures 4A, B**). In line with the cell numbers detected by flow cytometric analyses, Phl p 5^+^ B cells were also detected by immunofluorescence microscopy with the highest frequency in the B-cell zone of the spleen and to a lesser extent in the B-T cell border regions of the white pulp (**Supplementary Figure 4C**), while only a few Phl p 5^+^ B cells were detected in the thymus tissue (**Supplementary Figure 4D**).

Phl p 5-specific IgG_1_ was absent in the Phl p 5^+^ B cell-treated mice until week 22, after which it started to rise slowly. In contrast, the control group that received no cells developed regular Phl p 5-specific IgG_1_ responses immediately after the first sensitization at week 4, which were boosted after subsequent immunizations (**Figure 1D**). Importantly, no Phl p 5-specific IgG_1_ responses were detectable in Phl p 5^+^ B cell-treated mice until week 22. Similar to IgG_1_, Phl p 5-specific IgE was absent until weeks 18/22 (**Figure 1E**). The effect was specific to Phl p 5, as IgG_1_ and IgE responses specific to the control allergen, Bet v 1, were regularly observed immediately after the first sensitization (even though they occurred with some delay due to rapamycin/anti-CD40L treatment) (**Figures 1F, G**). Similar data were obtained in additional groups of mice with shorter follow-ups (**Supplementary Figures 5A–E**).

### 3.4 Adoptive transfer of Phl p 5^+^ B cells significantly reduces Phl p 5-induced allergic airway inflammation

**Figure 2:**
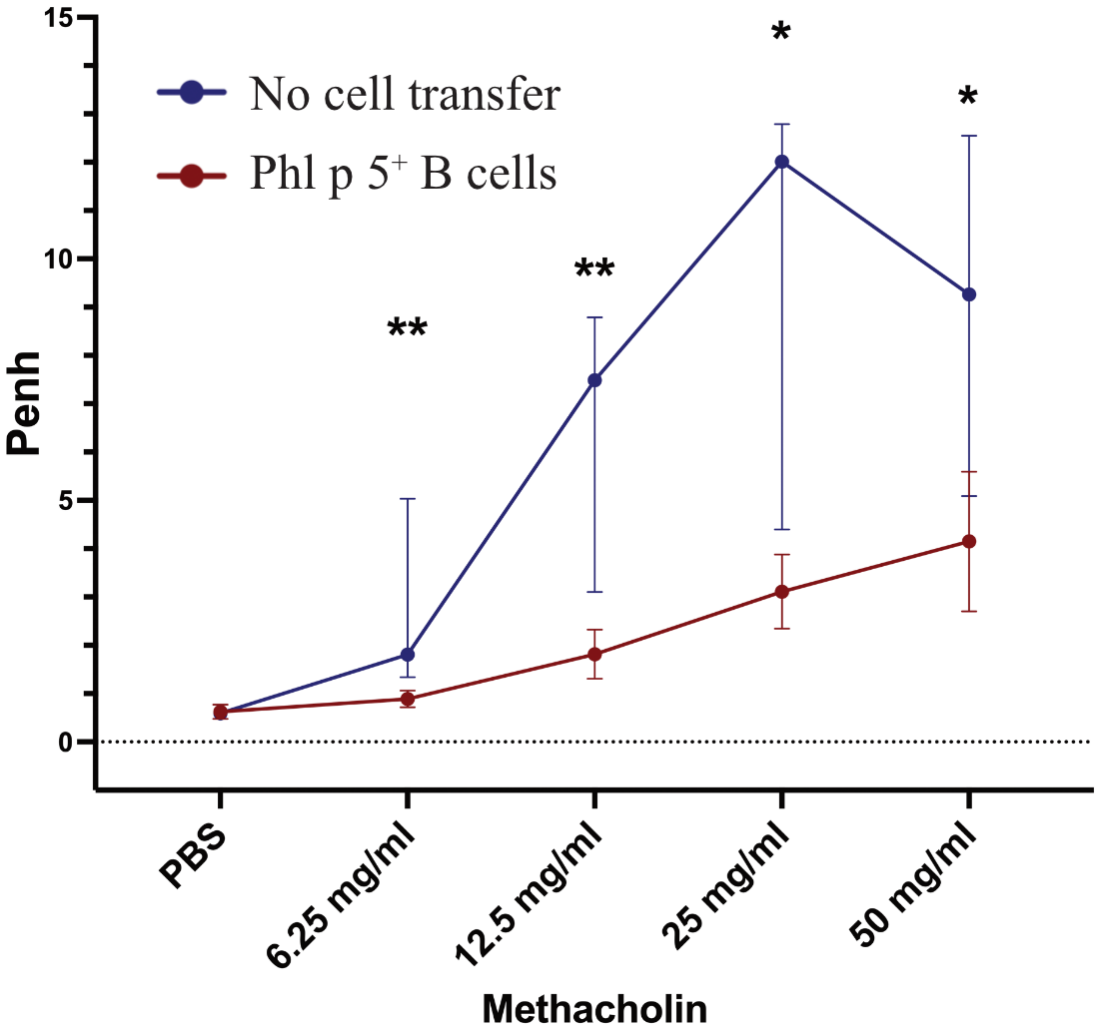
Phl p 5^+^ B cell therapy prevents allergic airway inflammation. Penh (Y-axis) in response to increasing concentrations of methacholine (X-axis) was measured by WBP in mice treated with Phl p 5^+^ B cell therapy [Phl p 5^+^ B cells, (n=6)] and as a control in mice that received no cells [no cell transfer (n=6)]. (Data from one experiment) The medians, interquartile range, and P values of Penh are shown.(* p<0.05; ** p <0.01).

Allergic sensitization to pollen poses a substantial risk for the development of allergic asthma (39). Therefore, we tested whether Phl p 5-induced allergic airway inflammation could be avoided by Phl p 5^+^ B cell transplantation. Lung function was measured at different time points in two groups of mice that received Phl p 5^+^ B cells. One measurement was performed when no Phl p 5-specific serum IgE levels were detectable (week 16), while the second group of mice was tested after the onset of a Phl p 5-specific IgE response (week 39). For this purpose, mice of the Phl p 5^+^ B cell group and the no cell transfer group received three intranasal applications of Phl p 5, followed by a challenge with increasing doses of methacholine and assessment of lung function by determining the enhanced pause in breathing (Penh) using WBP. We found a significantly lower Penh at all methacholine concentrations (6.25 – 50mg/ml) in the Phl p 5^+^ B cell group compared to the no cell transfer group at week 16, when mice had received Phl p 5 intra-nasally before the challenge (data not shown). Remarkably, this effect was maintained 39 weeks after the initial cell transfer, even though Phl p 5-specific IgE levels had already started to rise at this time point, suggesting that the tolerizing effect of Phl p 5^+^ B cell transplantation is long-lasting with respect to asthma prevention (**Figure 2**). These results are in accordance with a significantly reduced Phl p 5-specific T cell proliferation observed in the Phl p 5^+^ B cell group compared to Phl p 5 sensitized controls without cell therapy (**Supplementary Figure 6**).

### 3.5 Robust suppression of immediate-type skin hypersensitivity lasting for more than 4 months in mice adoptively transferred with Phl p 5^+^ B cells

**Figure 3:**
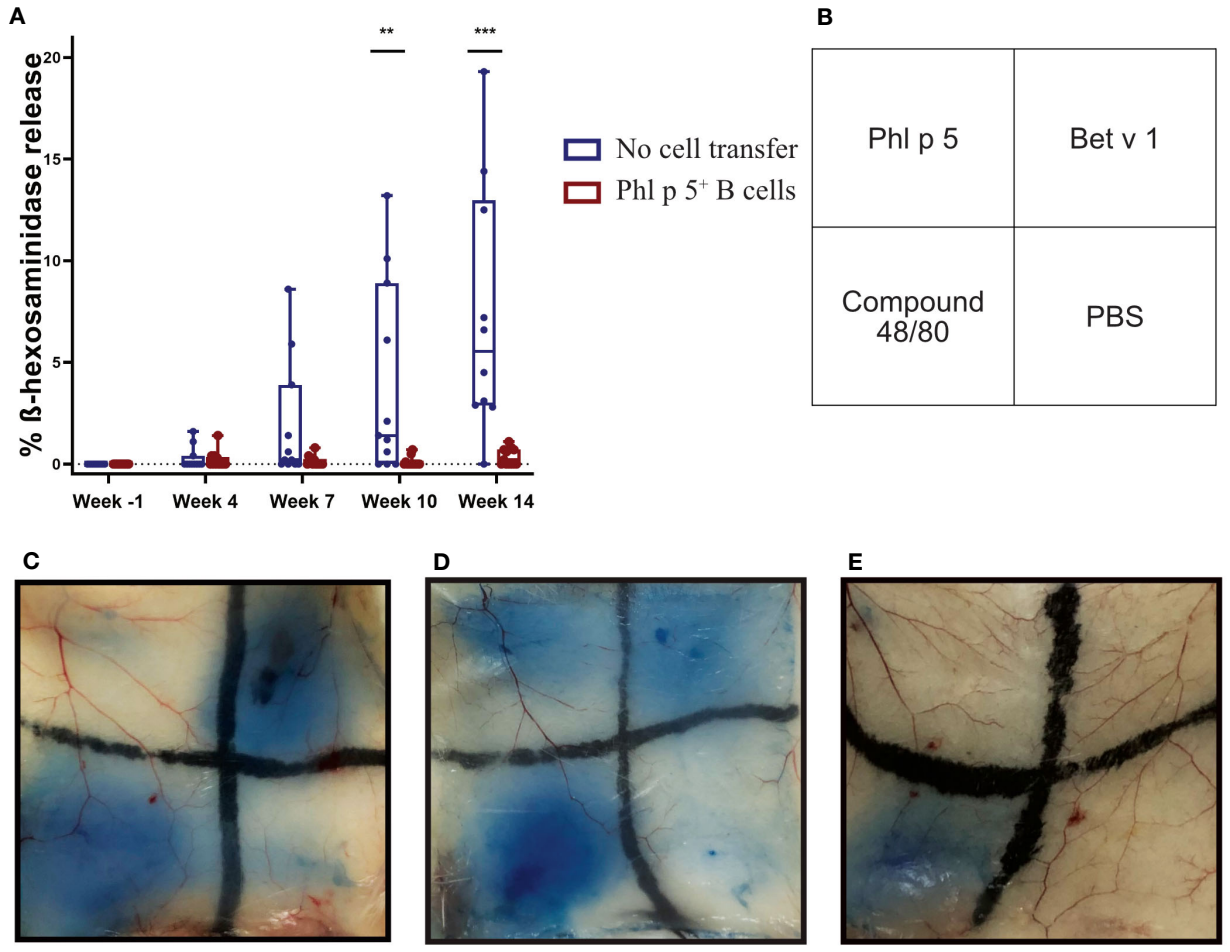
Phl p 5^+^ B cell therapy prevents allergen-specific basophil and mast cell responses *in vitro* and *in vivo*. **(A)** RBL cells were loaded with either sera from Phl p 5^+^ B cell-treated mice [Phl p 5^+^ B cell, (n=13)] or sera from mice from the no cell transfer group [no cell transfer, (n=11)] collected at the indicated time points (X-axis) and challenged with rPhl p 5. The percentages of total ß-hexosaminidase release are displayed on the y-axis as box plots, and significant P values are shown. Pooled data from three independent experiments and significant P values are shown. (** p<0.01; *** p <0.001) **(B)** Scheme of intradermal injection. **(C–E)** Representative skin sections of **(C)** mice treated with Phl p 5^+^ B cell therapy [Phl p 5+ B, (n=7)], **(D)** mice that received no cells, no immunosuppression, but immunization [referred to as no cell transfer, (n=2)], and **(E)** naive mice (n=4) are shown. Pooled data from two independent experiments are shown.

The effect of Phl p 5^+^ B cell therapy on Phl p 5-induced immediate-type hypersensitivity reactions was investigated in further experiments. For this purpose, mice were treated according to the experimental scheme (**Supplementary Figure 5A**), and the lack of Phl p 5-specific IgE in the Phl p 5^+^ B cell group at week 14 was further investigated in an RBL degranulation assay by measuring the activation and mediator release of effector cells *in vitro*. Sera from Phl p 5^+^ B cell mice and the no cell transfer control group mice were loaded onto RBL cells and stimulated with rPhl p 5. No Phl p 5-induced mediator release was detected (up to week 14) when cells were loaded with sera from the Phl p 5^+^ B cell-treated group, while sera from the control group induced a Phl p 5-specific release, starting with sera obtained at week 7 and reaching significance with sera obtained at week 10 (**Figure 3A**).

Next, we asked the question of whether Phl p 5-specific IgE antibodies, although not detectable in the serum of mice, were bound to the FcεRI receptor on mast cells. To this end, type I immediate hypersensitivity reactions to Phl p 5, Bet v 1, and PBS as a negative control and the mast cell activator compound 48/80 as a positive control were tested in the skin of Phl p 5^+^ B cell-treated mice in the no cell transfer and naive groups of mice (**Figure 3B**). The group of Phl p 5^+^ B cell-transplanted mice did not show any sign of mast cell degranulation after intradermal challenge with Phl p 5, but a positive reaction to challenge with the control allergen Bet v 1 (**Figure 3C**). In contrast, sensitized mice without cell therapy showed positive skin reactions to challenge with both allergens (**Figure 3D**), while no skin reaction was observed in naive BALB/c mice. (**Figure 3E**). From these results, we concluded that Phl p 5-specific IgE responses were specifically absent in the blood and tissue of mice who had received Ph l p 5-specific cell therapy.

### 3.6 Administration of anti-CTLA4 or anti-PD1 antibodies three weeks after cell therapy does not break tolerance to Phl p 5

Regulatory mechanisms play an important role in maintaining immunological self-tolerance and homeostasis (40). Previous studies have shown that rapamycin and anti-CD40L promote the generation of regulatory T cells (Tregs) in certain experimental settings (41). Therefore, we wanted to assess whether T regulatory mechanisms are important for the maintenance of tolerance in our model. The co-inhibitory receptor cytotoxic T lymphocyte antigen 4 (CTLA4) is constitutively expressed on Tregs. Controlling CD80/86 expression on antigen-presenting cells (APC) plays an important role in Treg-mediated suppression (42–44). Another co-inhibitory receptor, programmed cell death protein 1 (PD-1), is highly expressed on Tregs (45) and other cell subsets, including T follicular helper cells. Under physiological conditions, PD-1 expression in combination with PD-L1 maintains the necessary balance between T cell activation, tolerance, and immune-mediated tissue damage. A lack of PD-1 expression on Tregs, therefore, leads to different autoimmune conditions, whereas upregulation of PD-1 on Tregs affects tumor surveillance (46). In our setting, we tested whether anti-CTLA4 and anti-PD1 antibodies, administered three weeks after cell therapy with Phl p 5^+^ B cells, had an impact on Phl p 5-specific tolerance induction.

**Figure 4:**
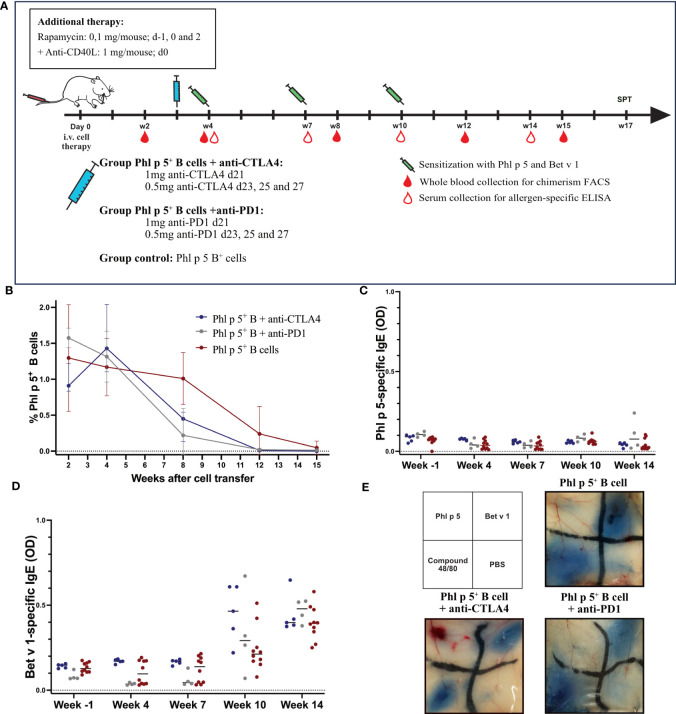
Phl p 5-specific tolerance persists after anti-CTLA4 or anti-PD1 administration. **(A)** Mouse experimental scheme of B cell therapy in naïve BALB/c recipient mice (6-8 weeks), pre-treated with anti-CD40L (1mg on day 0) and a short course of rapamycin (0.1 mg on days -1, 0, and 2) prior to cell transfer from a Phl p 5-transgenic mouse. In addition, mice received either anti-CTLA4 or anti-PD1 at the indicated time points. Mice were sensitized to Phl p 5 and Bet v 1 at weeks 4, 7, and 10. Serum and peripheral blood were collected at the indicated time points. **(B)** Percentages of Phl p 5 expressing B cells in peripheral blood were analyzed by flow cytometric analyses in recipient mouse groups (Phl p 5^+^ B +anti-CTLA4, n=5, Phl p 5^+^ B +anti-PD1, n=4 and Phl p 5^+^ B cells, n=10) at multiple time points (X-axis). The median and interquartile range of the percentage of Phl p 5^+^ cells are shown on the Y-axis. **(C)** Phl p 5-specific and **(D)** Bet v 1-specific IgE levels in sera from mice treated as indicated in **(A)** are shown. Median OD levels (Y-axis) are shown at the indicated time points (X-axis). Results are displayed as dot blots, and medians are shown. **(E)** Scheme of intradermal injection and representative skin sections of Phl p 5^+^ B cells (n=7), Phl p 5^+^ B cells +anti CTLA4 (n= 5) and Phl p 5^+^ B cells + anti-PD1 (n=4) mice are shown. (Data from one experiment).

As shown in **Figure 4A**, BALB/c recipient mice were treated with isolated Phl p 5 ^+^ B cells together with rapamycin and anti-CD40L. In addition, groups of mice were treated with either anti-CTLA4 monoclonal antibody (mAB) (1mg d21; 0.5 mg d23, 25, and 27) or with anti-PD1 (1 mg d21; 0.5 mg d23, 25, and 27) and compared to a Phl p 5^+^ B cell group (**Figure 4A**).

Phl p 5- expressing B cells were followed in the peripheral blood by flow cytometric analyses, and Phl p 5^+^ B cell chimerism ranged from 0.91-1.58% (median values) without significant difference. Thus, all mice transplanted with Phl p 5^+^ B cells developed a clear B cell chimerism at this time point. The B cell chimerism in the Phl p 5^+^ B cell-treated mice that received anti-CTLA4 or anti-PD1 declined more rapidly compared to the Phl p 5^+^ B control and was almost gone by week 12 compared to the control, where B cell chimerism remained detectable until week 15 (**Figure 4B**). However, the exact time point of loss of B cell chimerism could not be determined in our experiment. Despite the earlier loss of the B cell chimerism in anti-CTLA4 or anti-PD1-treated mice, they stayed tolerant throughout the follow-up period (week 14), as shown by the absence of Phl p 5-specific IgE in the serum (**Figure 4C**). Notably, the humoral response to the control allergen Bet v 1 remained unchanged between the different groups (**Figure 4D**).

Additionally, skin mast cells were analyzed for their anaphylactic activity. None of the groups showed a positive skin reaction after the intradermal challenge with Phl p 5. In contrast, the three groups of mice showed clear-cut positive reactions to challenge with the control allergen Bet v 1 (**Figure 4E**). Taken together, our data suggest that selective elimination of either the CTLA4- or PD1-mediated regulatory pathway does not abrogate the maintenance of tolerance to the B cell-borne allergen.

## 4 Discussion

In this study, we developed a protocol for tolerance induction to a major grass pollen allergen (Phl p 5) based on the transfer of Phl p 5-expressing B cells to naive mice. Despite repeated allergen exposure and challenge, Phl p 5^+^ B cell-treated mice showed a complete lack of allergen-specific IgE and IgG_1_ responses until week 22, together with effective prevention of mast cell degranulation by week 17 and protection from T cell-mediated asthma by week 39.

In the setting of allogeneic transplantation, the transfer of CD4^+^/CD8^+^ T cells (47, 48) and bone marrow-derived stem cells differentiated into plasmacytoid DC, but not the transfer of B cells (48), has been shown to induce tolerance to donor grafts (49). In the present allergen-specific tolerance study, transfer of Phl p 5^+^ B cells induced a low level of B cell chimerism in recipient mice, which was accompanied by a lack of Phl p 5-specific IgE and IgG_1_ responses despite repeated allergen exposure at weeks 4, 7, and 10. Notably, the percentage of chimerism was lower than that observed after Phl p 5^+^ bone marrow cell transplantation (31) and disappeared in the peripheral blood four months after cell transfer, as was expected since no HSC was transferred. Nevertheless, mice remained tolerant until week 26 and showed significantly better lung function until week 39. In the field of transplantation, the minimum level of chimerism necessary for tolerance induction has not been firmly established, but very low levels may be sufficient (50), and even transient chimerism can induce lasting tolerance (51, 52). Transfer of a CD4^+^/CD8^+^ T cell mixture and – to some degree – of Flt3L-stimulated DC also led to tolerance to Phl p 5. We focused on B cells as a cell therapy candidate because of their efficacy and because clinical translation appears to be particularly feasible with this lymphocyte subset.

Based on our current results, we can only speculate whether peripheral or central tolerance mechanisms are involved in our model. B cells were followed in the different lymphatic organs and were found to home in high numbers in the spleen, which is known to filter blood-borne antigens and has also been described as a possible site for peripheral tolerance induction (53). Interference with two important molecules, CTLA4 and PD1, both involved in Treg function, had no effect on our model, suggesting that Tregs are not critically involved in allergen-specific tolerance induction. However, the inactivation of Tregs may have been incomplete. We do not believe that mast cell mediators contributed to tolerance induction as described in rodent models of transplantation (54, 55). According to these findings, the simultaneously occurring Bet v 1-induced allergic inflammation could have negatively interfered with tolerance to Phl p 5 (56), which was not the case in our studies. In principle, regulatory B cells (Breg) could also play a role, as their anti-inflammatory functions have been described in different disease models, including allergic inflammation and transplantation (57). However, the activation of CD40 is believed to be important for Breg generation, and we found the combination of rapamycin and anti-CD40L to be more efficient than rapamycin and CTL4Ig in our model. B cells were also found at low levels in the thymus, the site where central tolerance induction is initiated. Previous work has shown the importance of B cell homing to the thymus. Such cells are allowed to enter the thymus and have been shown to present antigens to developing T cells (58–60). Therefore, central tolerance induction may also be a possible mechanism operating in our study setting, but further experiments beyond the scope of the current work are needed to clarify this issue.

Humoral tolerance to Phl p 5, as shown by the complete absence of allergen-specific IgE and IgG_1_, was particularly long-lasting and only started to wane after approximately 5 months. Interestingly, and in contrast to the rising allergen-specific antibody levels observed from week 22 onwards, B cell-treated mice showed protection against lung hyperreactivity even 39 weeks after cell transfer. It is tempting to speculate that repeated/additional administration of the cell therapy could be considered and could further extend the tolerogenic effect.

Importantly, the developed cell therapy protocol does not require any cytotoxic or myelosuppressive preconditioning, which is part of many previous tolerance induction models (28, 61). The tolerance-inducing protocol established here includes the administration of immunosuppressive drugs, but these are mild and only used for a short period of time around the time of adoptive cell transfer. The mTOR inhibitor rapamycin is approved for immunosuppression in organ transplantation (62). Second-generation anti-CD40L antibodies are in clinical development and appear to be safe, without the deleterious thromboembolic side effects that had been seen with earlier versions of anti-CD40L antibodies (63, 64). Thus, the described protocol represents a significant step forward toward the long-term vision of the clinical application of cell-based prevention of allergy in terms of both efficacy and safety.

In the experiments presented herein, donor cells were collected from a transgenic mouse model that expressed the allergen on the cell surface of all body cells. The next step would be to use autologous cells expressing the allergens in various ways. In this sense, the use of RNA expression systems has recently become popular for the expression of heterologous molecules, such as tumor and viral proteins (65, 66). Another attractive alternative could be the chemical coupling of allergens to the cell surface of, for instance, peripheral blood-derived cell types such as B cells (67). Both approaches may be feasible, as tolerance induction seems to occur within a short period after cell transfer. To further improve safety, allergen-derived peptides or hypoallergenic allergen derivatives could be used, avoiding the risk of sensitization to the allergen (6, 68). Allergic sensitization takes place early in life, which pushes the ideal time point for allergen-specific prophylaxis to an early phase after birth. Therefore, cord blood cells could serve as a cell source for CD19^+^ B cells, especially since they contain a high frequency of B cells (69). Furthermore, shifting the intervention to an earlier time point could theoretically even prevent the use of immunosuppressive drugs, as has previously been shown in a transplant model (70).

In conclusion, our results demonstrate a robust and long-lasting prophylactic tolerance induction that prevents Phl p 5-specific T and B cell responses. The long follow-up of 39 weeks highlights the use of cell therapy under a simple and short-term immunosuppressive regimen without irradiation and is unique among other tolerance protocols. This offers a long-term vision of how cell therapy could be used in tolerance induction to prevent IgE-mediated allergy.

## Data availability statement

The raw data supporting the conclusions of this article will be made available by the authors, without undue reservation.

## Ethics statement

The animal study was approved by Austrian Federal Ministry of Education, Science, and Research. (Permission number GZ: GZ 2021-0.276.441 and BMWFW-66.009/0028-WF/V/3b/2015). The study was conducted in accordance with the local legislation and institutional requirements.

## Author contributions

LP: Investigation, Methodology, Visualization, Writing – original draft, Writing – review & editing. AW: Investigation, Writing – review & editing. VK: Investigation, Writing – review & editing. UB: Investigation, Writing – review & editing, Methodology, Funding acquisition. BK: Investigation, Writing – review & editing. RS: Investigation, Writing – review & editing. KM: Investigation, Writing – review & editing. JM: Investigation, Writing – review & editing. ER: Investigation, Writing – review & editing. NP: Investigation, Writing – review & editing. BB: Resources, Writing – review & editing. HS: Writing – review & editing, Methodology. WP: Writing – review & editing, Methodology. RV: Writing – review & editing, Conceptualization, Funding acquisition. BL: Conceptualization, Funding acquisition, Investigation, Methodology, Project administration, Writing – review & editing, Supervision, Writing – original draft. TW: Conceptualization, Funding acquisition, Methodology, Project administration, Writing – review & editing, Supervision, Writing – original draft.

## Supplementary material

Supplementary Figure 1 Expression of Phl p 5 and GFP in immune cells of the Phl p 5-transgenic mouse. Dual parameter contour plots show expression levels of Phl p 5 (Y-axis) and GFP (X-axis) in a naive BALB/c control mouse and whole blood, lymph nodes, and spleen of a Phl p 5-transgenic mouse. Cells were gated for lymphocytes, singlets, live cells, and CD45.2. The numbers in the quadrants indicate the percentage of positive cells.

Supplementary Figure 2 Characterization of Phl p 5-transgenic cells. **(A)** Pseudocolor bivariate density plots of CD19^+^ B cells gated on lymphocytes, singlets, live cells, CD45.2, and GFP showed a purity of 96.5%. **(B)** Bright-field and fluorescence microscopy pictures of a B220^+^ B cell from splenocytes of a Phl p 5^+^ mouse. GFP is expressed cytoplasmically (green), and Phl p 5 is expressed on the cell surface (yellow).

Supplementary Figure 3 Phl p 5^+^ B cell therapy with rapamycin and CTLA4Ig does not induce specific tolerance to Phl p 5. **(A, B)** Phl p 5-specific and **(C, D)** Bet v 1-specific IgG_1_ and IgE levels in sera from mice treated with Phl p 5^+^ B cells + anti-DC40L (n=3), Phl p 5^+^ B + CTLA4Ig (n=3) and no cells (n=3) are shown. Median OD levels (Y-axis) are shown at indicated time points (X-axis) are shown as dot blots with medians. (Data from one experiment).

Supplementary Figure 4 Injected Phl p 5^+^ B cells colonize different organs. **(A)** Total cell counts of Phl p 5^+^ B cells in spleen, thymus, lymph nodes (axillary, brachial, and inguinal), and bone marrow (femur, tibia, and humerus) (y-axis) at indicated time points (x-axis) after Phl p 5^+^ B cell transfer. **(B)** Percentage of live cells within the Phl p 5^+^ B cell fraction (y-axis) at different time points (x-axis). In **(A)** and **(B)**, the medians and interquartile ranges are shown. **(C, D)** Representative immunofluorescence staining of the spleen **(C)** and thymus **(D)** 7 days after Phl p 5^+^ B cell transfer. B220^+^ B cells are visible in red, and CD3^+^ T cells are visible in yellow or cyan. White arrows mark GFP-positive B cells (green). Scale bars are shown in the left corners. All cells were isolated from mice treated with 10 x 10^6^ Phl p 5^+^ B cells together with anti-CD40L (1 mg on day 0) and rapamycin (0.1mg on days -1, 0, and 2). Four mice were sacrificed per time point, and flow cytometric analysis was performed on cells from each individual mouse. Five additional mice were sacrificed on day 7 for immunofluorescence staining. Frozen sections of the spleen and thymus were blocked with 5% BSA in 1xPBS/0.1% Tween 20. Cell nuclei were stained with DAPI (4′,6-diamidino-2-phenylindole) 1 µg/ml (BioLegend, San Diego, CA, USA) (blue). B cells were stained with anti-CD45R (B220) IgG antibody (Thermo Fisher Scientific, MA, USA), and bound IgG was visualized using secondary antibodies labeled with AF633 (Thermo Fisher Scientific, MA, USA) (red). T cells were stained with an anti-CD3 IgG antibody, and bound IgG was visualized by using secondary antibodies labeled with AF555 (Abcam, Cambridge, UK) (yellow or cyan). Phl p 5-expressing B cells were identified by intracellular GFP expression. Specimens were analyzed on a confocal LSM700 microscope (Zeiss, Oberkochen, Germany) using the ZEN software (Zeiss, Oberkochen, Germany) and Fiji software. Magnification 20x. Pooled data from two independent experiments are shown.

Supplementary Figure 5 Phl p 5^+^ B cell therapy with rapamycin and anti-CD40L antibody leads to specific tolerance induction. **(A)** Mouse experimental scheme of cell therapy in naive recipient mice. BALB/c recipient mice (6-8 weeks) received pre-treatment with anti-CD40L (MR1; 1 mg on day 0) and a short course of rapamycin (0.1mg on days -1, 0, and 2). prior to cell transfer from a Phl p 5-transgenic mouse. A positive control group received rapamycin plus CTLA4Ig (0.5 mg on day 2) according to a previously established protocol using bone marrow cells (31). Mice were immunized with aluminum hydroxide-adsorbed Phl p 5 and Bet v 1 at weeks 4, 7, and 10. Serum and whole blood were collected at the indicated time points. 10 ug rPhl p 5 was administered intranasally on days -3, -2, and -1 before WBP. **(B, C)** Median Phl p 5-specific IgG_1_ and IgE levels (Y-axis) in sera of mice treated with either 10 x 10^6^ Phl p 5^+^ B cells (Phl p 5 B^+^, (n = 13)) or 2 x 10^7^ unseparated bone marrow cells isolated from Phl p 5^+^ transgenic mouse donors (Phl p 5^+^ BM, n=6). For control purposes, groups that received no cells, no immunosuppression, but immunization (referred to as no cell transfer, (n=11)) were included. As an additional control group, mice received no cells, pre-treatment with costimulation blockade anti-CD40L, a short course of rapamycin, and the immunization (referred to as no cell transfer + IS, (n=3)). Data are shown at different time points (X-axys). **(D, E)** Bet v 1-specific IgG_1_ in the groups of mice described in **(B, C)**. Pooled data from three independent experiments are shown. Results are presented as box plots and significant P values are indicated. (** p<0.01; *** p <0.001; **** p <0.0001).

Supplementary Figure 6 T cell proliferation in response to Phl p 5 and Bet v 1 as measured by thymidine incorporation assay. Splenocytes were isolated from Phl p 5 and Bet v 1 sensitized mice (n=5), from mice having received cell therapy (n=4), and from naive control mice (n=2) and incubated in the presence of Phl p 5, Bet v 1, ConA, or medium before adding ^3^H-labeled thymidine for 16 hours. Cell proliferation was measured on a beta counter, as previously described in (31). **(A)** The proliferation response shown as stimulation indices (y-axis) for Phl p 5 (x-axis). **(B)** Proliferation response is shown as stimulation indices (y-axis) for Bet v 1 (x-axis). Pooled data from two independent experiments are shown. Results are presented as box plots and significant P values are indicated. (* p<0.05; ns, not significant). *Note*: data from the control group were also partially shown in (31).

Supplementary Table 1 Immunization and treatment protocol. Cell therapy, rapamycin, and antibody treatment were performed as shown in the table. Mice were sensitized with Phl p 5 and Bet v 1. Whole-body plethysmography (WBP) and cutaneous hypersensitivity testing (SPT) to Phl p 5 were performed at the given time points.
