# Supplementary figures and images for: Adoptive transfer of allergen-expressing B cells prevents IgE-mediated allergy
Source: Front Immunol. 2023 Nov 23;14:1286638. doi: 10.3389/fimmu.2023.1286638 (PMC10703460; doi:10.3389/fimmu.2023.1286638)

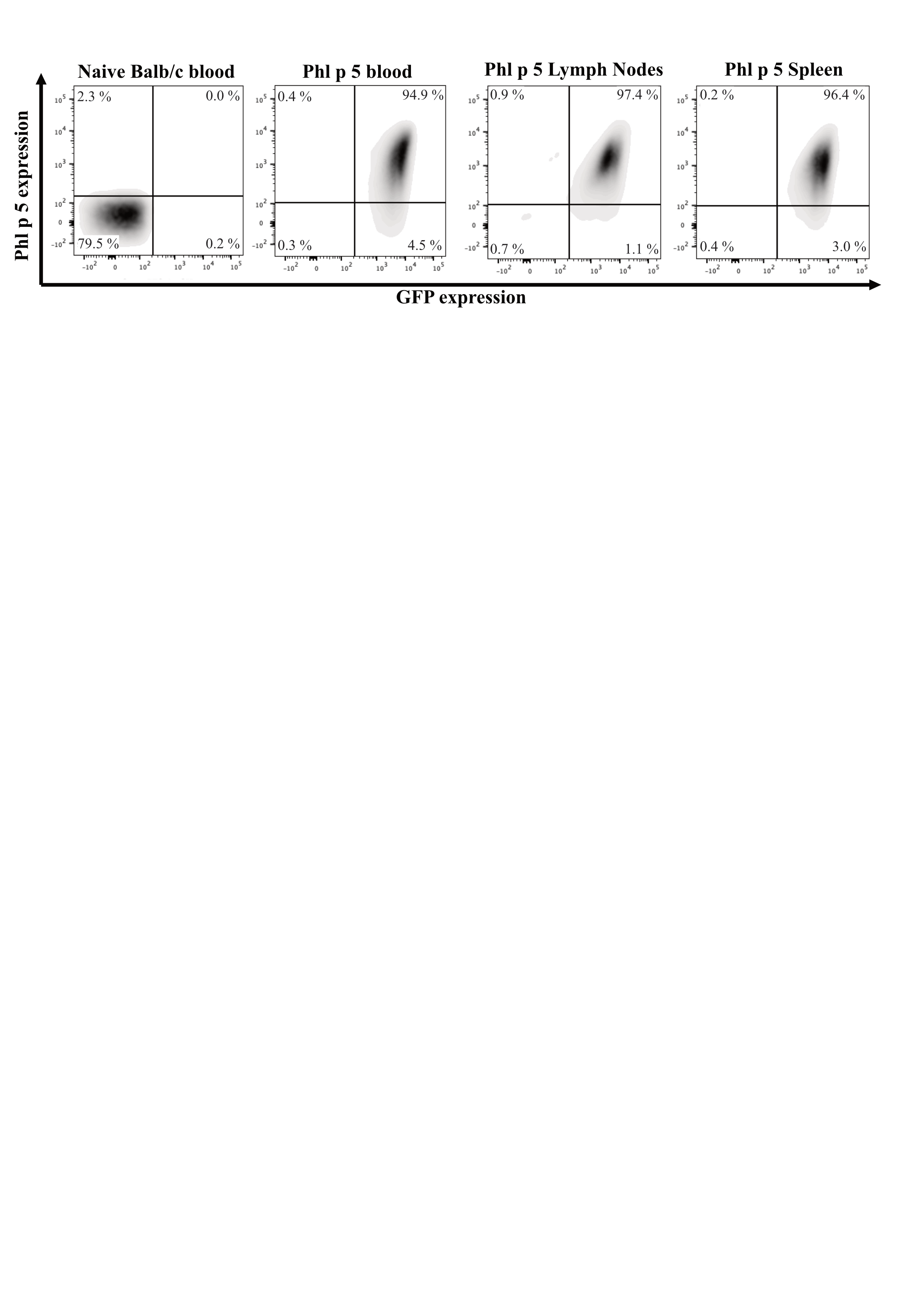

Supplement: Supplementary Figure 1 — Expression of Phl p 5 and GFP in immune cells of the Phl p 5-transgenic mouse. Dual parameter contour plots show expression levels of Phl p 5 (Y-axis) and GFP (X-axis) in a naive BALB/c control mouse and whole blood, lymph nodes, and spleen of a Phl p 5-transgenic mouse. Cells were gated for lymphocytes, singlets, live cells, and CD45.2. The numbers in the quadrants indicate the percentage of positive cells. [file Image_1.tiff]

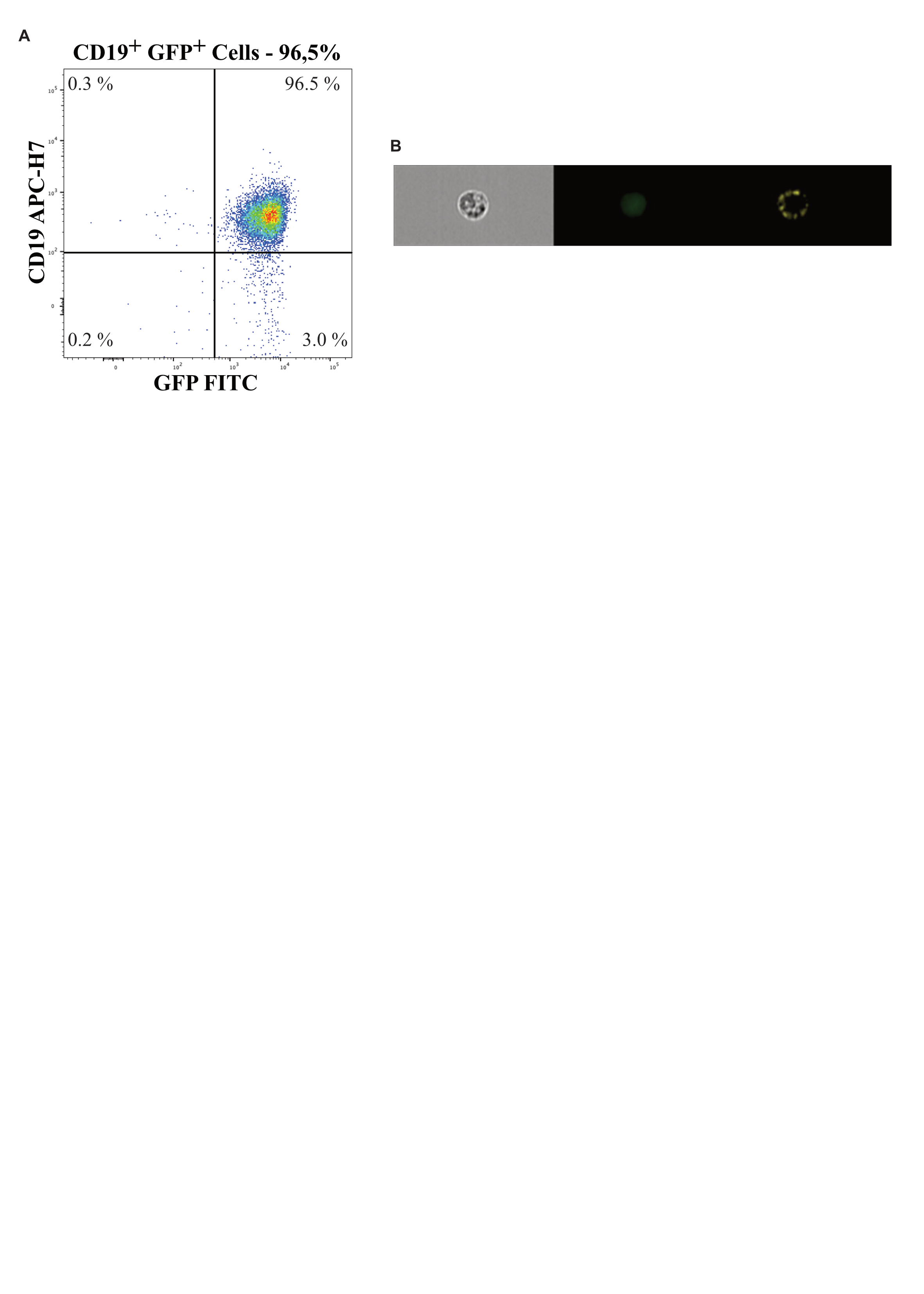

Supplement: Supplementary Figure 2 — Characterization of Phl p 5-transgenic cells. (A) Pseudocolor bivariate density plots of CD19+ B cells gated on lymphocytes, singlets, live cells, CD45.2, and GFP showed a purity of 96.5%. (B) Bright-field and fluorescence microscopy pictures of a B220+ B cell from splenocytes of a Phl p 5+ mouse. GFP is expressed cytoplasmically (green), and Phl p 5 is expressed on the cell surface (yellow). [file Image_2.tiff]

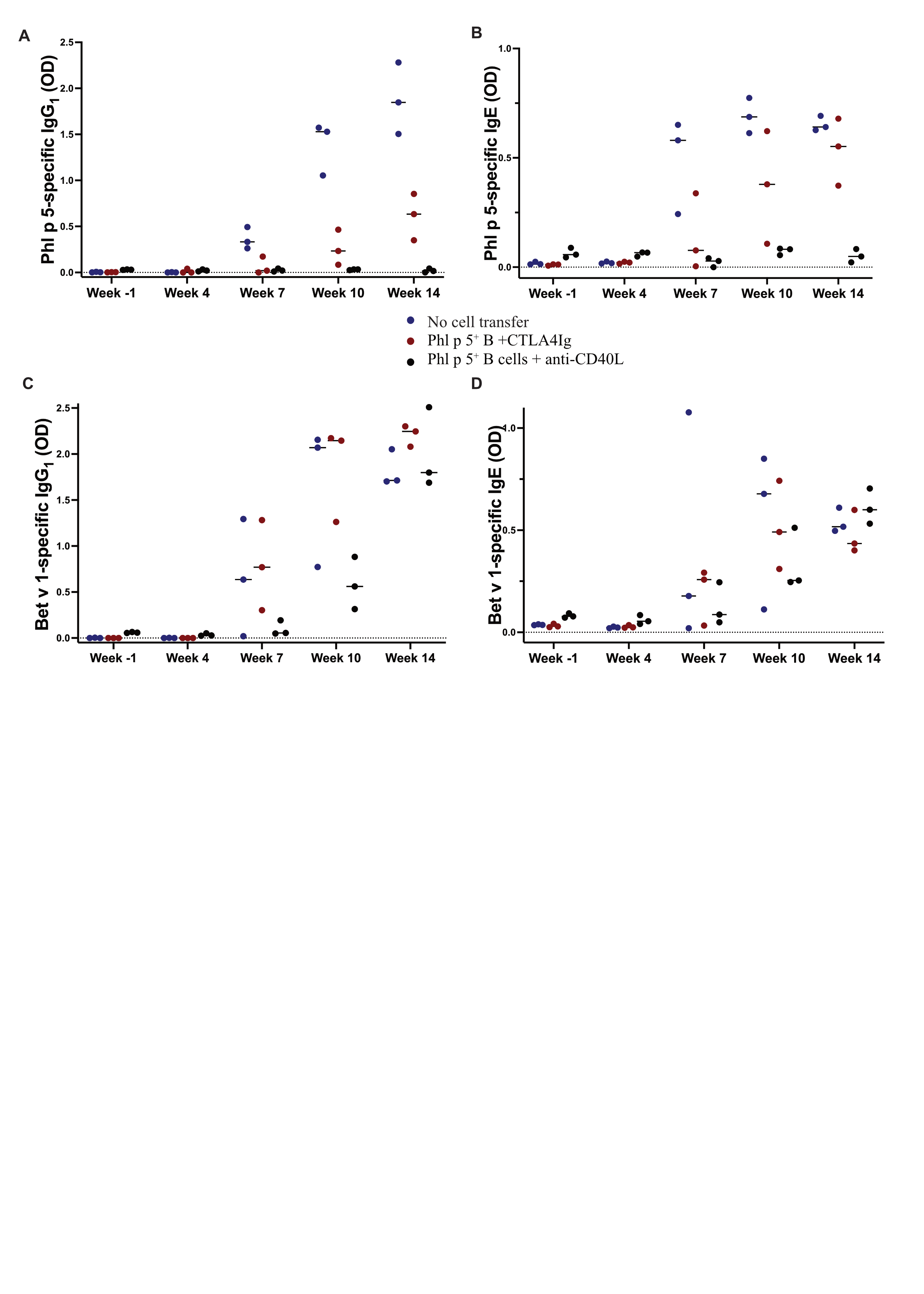

Supplement: Supplementary Figure 3 — Phl p 5+ B cell therapy with rapamycin and CTLA4Ig does not induce specific tolerance to Phl p 5. (A, B) Phl p 5-specific and (C, D) Bet v 1-specific IgG1 and IgE levels in sera from mice treated with Phl p 5+ B cells + anti-DC40L (n=3), Phl p 5+ B + CTLA4Ig (n=3) and no cells (n=3) are shown. Median OD levels (Y-axis) are shown at indicated time points (X-axis) are shown as dot blots with medians. (Data from one experiment). [file Image_3.tiff]

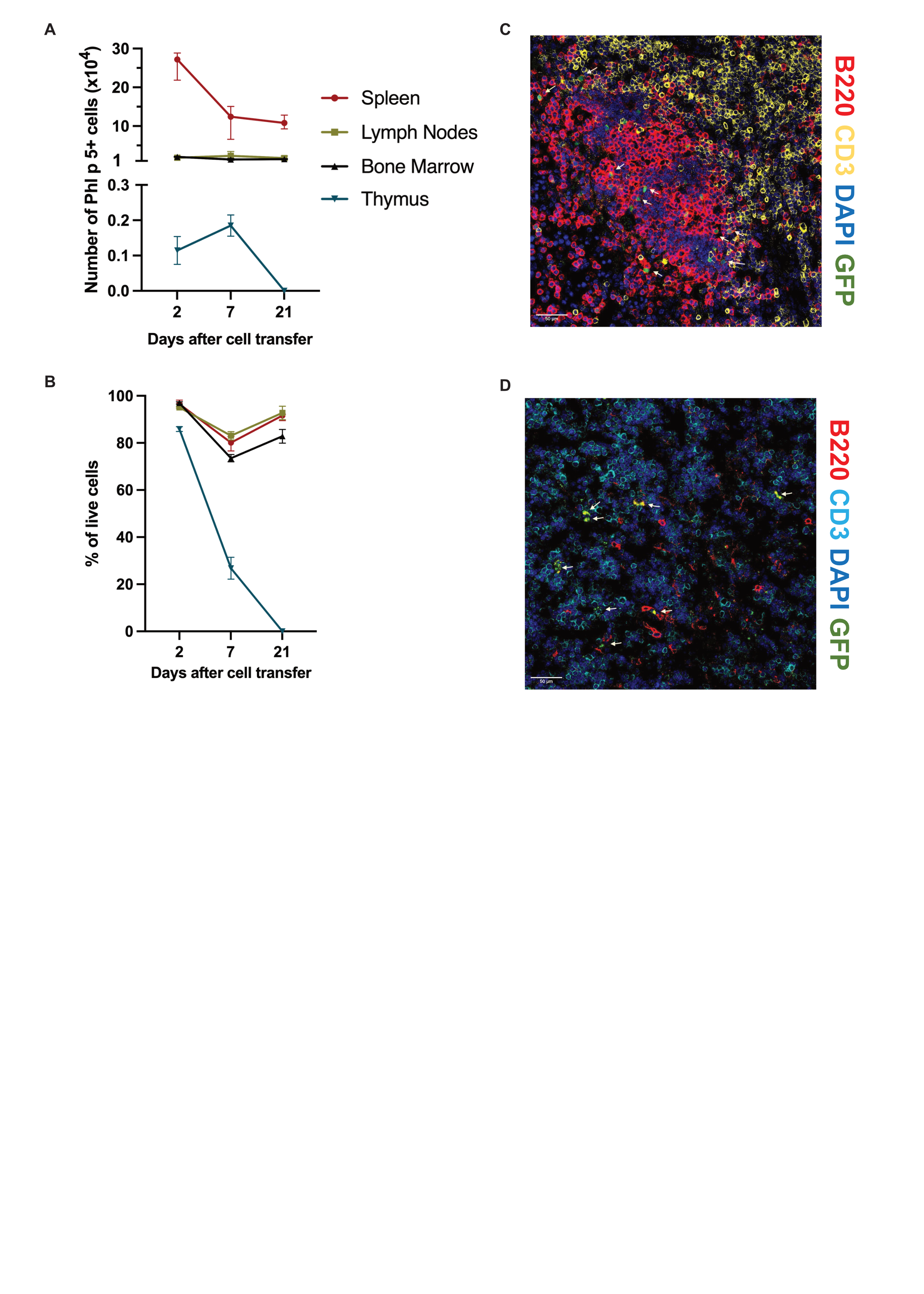

Supplement: Supplementary Figure 4 — Injected Phl p 5+ B cells colonize different organs. (A) Total cell counts of Phl p 5+ B cells in spleen, thymus, lymph nodes (axillary, brachial, and inguinal), and bone marrow (femur, tibia, and humerus) (y-axis) at indicated time points (x-axis) after Phl p 5+ B cell transfer. (B) Percentage of live cells within the Phl p 5+ B cell fraction (y-axis) at different time points (x-axis). In (A) and (B), the medians and interquartile ranges are shown. (C, D) Representative immunofluorescence staining of the spleen (C) and thymus (D) 7 days after Phl p 5+ B cell transfer. B220+ B cells are visible in red, and CD3+ T cells are visible in yellow or cyan. White arrows mark GFP-positive B cells (green). Scale bars are shown in the left corners. All cells were isolated from mice treated with 10 x 106 Phl p 5+ B cells together with anti-CD40L (1 mg on day 0) and rapamycin (0.1mg on days -1, 0, and 2). Four mice were sacrificed per time point, and flow cytometric analysis was performed on cells from each individual mouse. Five additional mice were sacrificed on day 7 for immunofluorescence staining. Frozen sections of the spleen and thymus were blocked with 5% BSA in 1xPBS/0.1% Tween 20. Cell nuclei were stained with DAPI (4′,6-diamidino-2-phenylindole) 1 µg/ml (BioLegend, San Diego, CA, USA) (blue). B cells were stained with anti-CD45R (B220) IgG antibody (Thermo Fisher Scientific, MA, USA), and bound IgG was visualized using secondary antibodies labeled with AF633 (Thermo Fisher Scientific, MA, USA) (red). T cells were stained with an anti-CD3 IgG antibody, and bound IgG was visualized by using secondary antibodies labeled with AF555 (Abcam, Cambridge, UK) (yellow or cyan). Phl p 5-expressing B cells were identified by intracellular GFP expression. Specimens were analyzed on a confocal LSM700 microscope (Zeiss, Oberkochen, Germany) using the ZEN software (Zeiss, Oberkochen, Germany) and Fiji software. Magnification 20x. Pooled data from two independent experiments are shown [file Image_4.tiff]

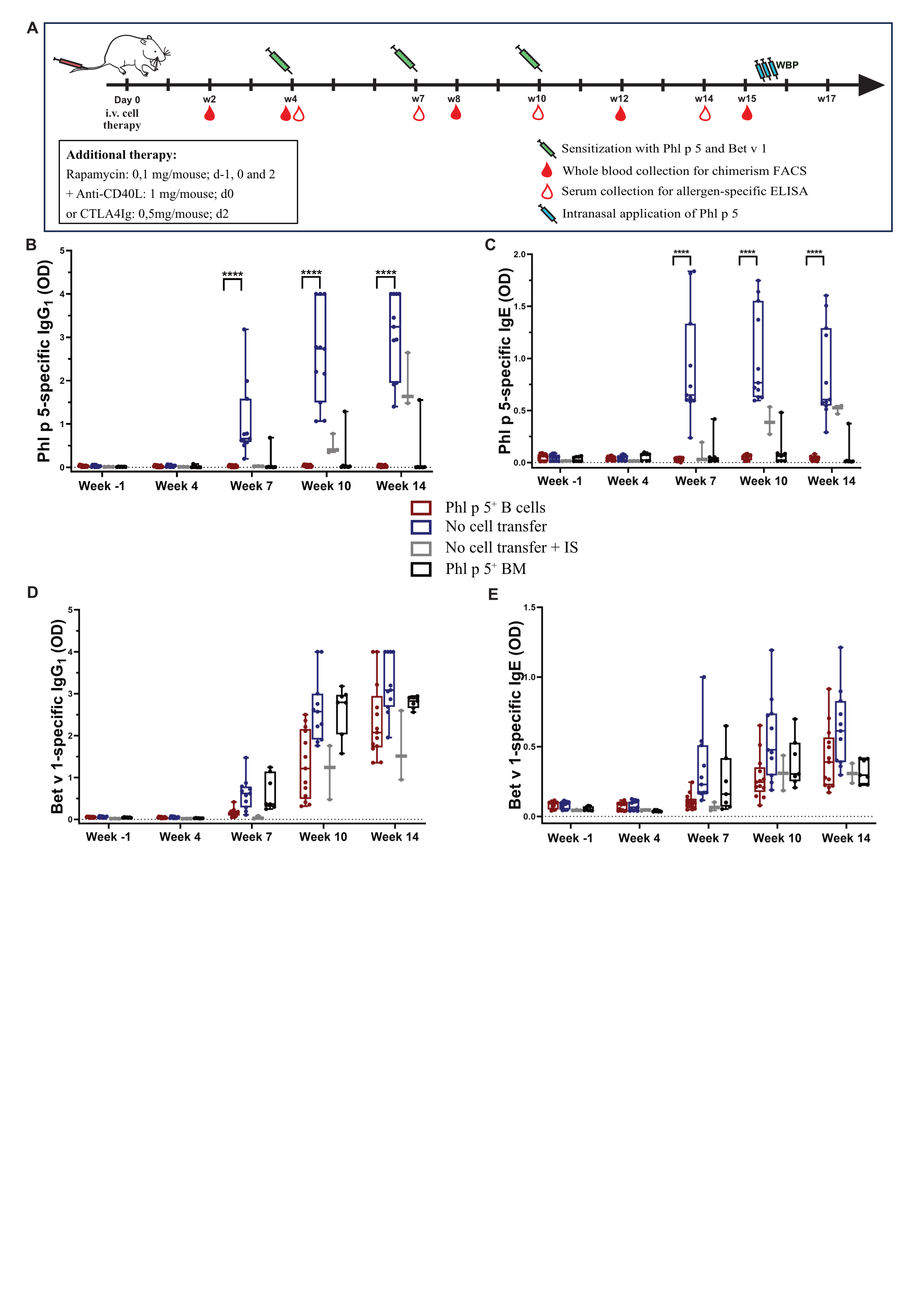

Supplement: Supplementary Figure 5 — Phl p 5+ B cell therapy with rapamycin and anti-CD40L antibody leads to specific tolerance induction. (A) Mouse experimental scheme of cell therapy in naive recipient mice. BALB/c recipient mice (6-8 weeks) received pre-treatment with anti-CD40L (MR1; 1 mg on day 0) and a short course of rapamycin (0.1mg on days -1, 0, and 2). prior to cell transfer from a Phl p 5-transgenic mouse. A positive control group received rapamycin plus CTLA4Ig (0.5 mg on day 2) according to a previously established protocol using bone marrow cells (31). Mice were immunized with aluminum hydroxide-adsorbed Phl p 5 and Bet v 1 at weeks 4, 7, and 10. Serum and whole blood were collected at the indicated time points. 10 ug rPhl p 5 was administered intranasally on days -3, -2, and -1 before WBP. (B, C) Median Phl p 5-specific IgG1 and IgE levels (Y-axis) in sera of mice treated with either 10 x 106 Phl p 5+ B cells (Phl p 5 B+, (n = 13)) or 2 x 10^7^ unseparated bone marrow cells isolated from Phl p 5+ transgenic mouse donors (Phl p 5+ BM, n=6). For control purposes, groups that received no cells, no immunosuppression, but immunization (referred to as no cell transfer, (n=11)) were included. As an additional control group, mice received no cells, pre-treatment with costimulation blockade anti-CD40L, a short course of rapamycin, and the immunization (referred to as no cell transfer + IS, (n=3)). Data are shown at different time points (X-axys). (D, E) Bet v 1-specific IgG1 in the groups of mice described in (B, C). Pooled data from three independent experiments are shown. Results are presented as box plots and significant P values are indicated. (** p<0.01; *** p <0.001; **** p <0.0001). [file Image_5.tiff]

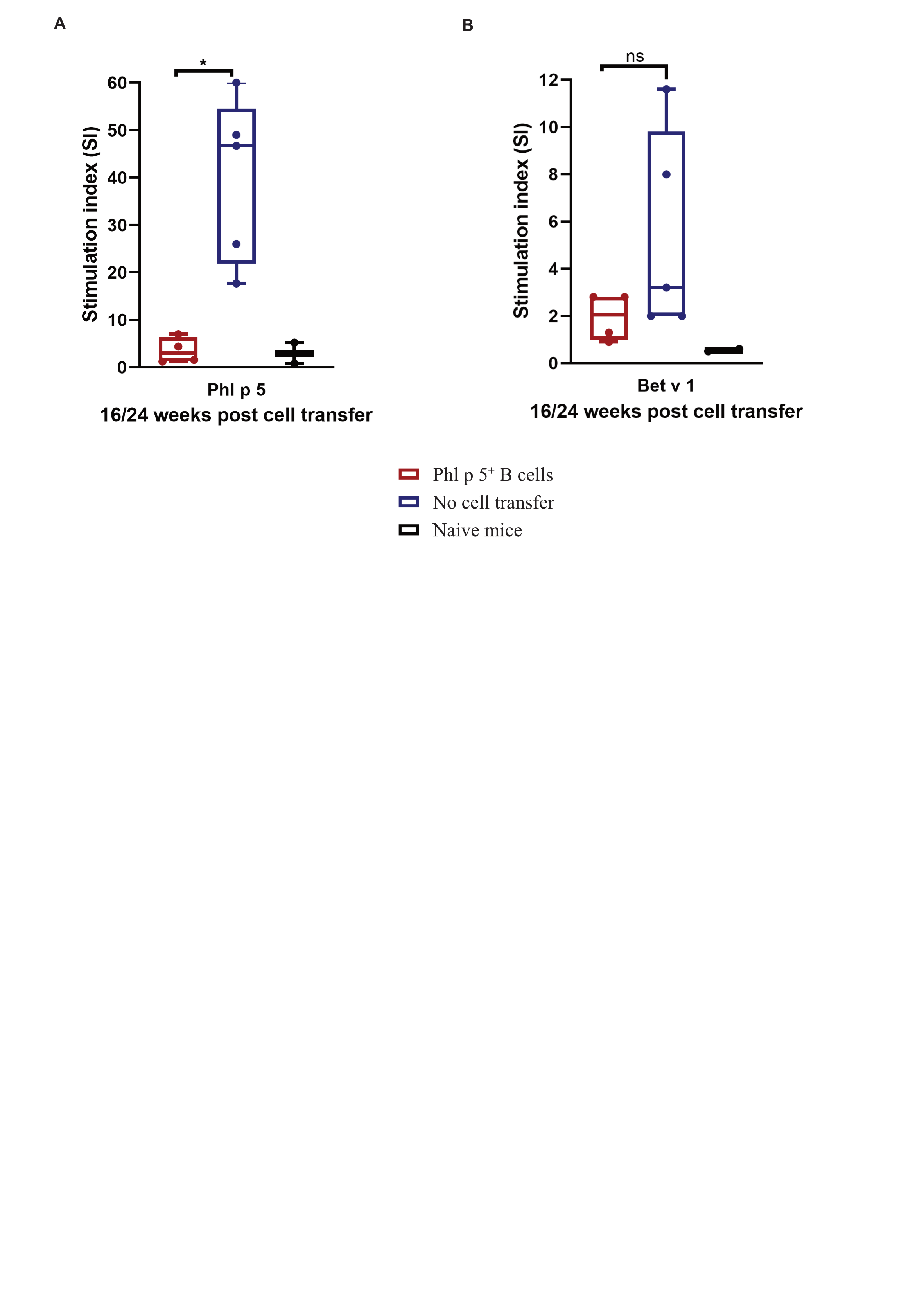

Supplement: Supplementary Figure 6 — T cell proliferation in response to Phl p 5 and Bet v 1 as measured by thymidine incorporation assay. Splenocytes were isolated from Phl p 5 and Bet v 1 sensitized mice (n=5), from mice having received cell therapy (n=4), and from naive control mice (n=2) and incubated in the presence of Phl p 5, Bet v 1, ConA, or medium before adding 3H-labeled thymidine for 16 hours. Cell proliferation was measured on a beta counter, as previously described in (31). (A) The proliferation response shown as stimulation indices (y-axis) for Phl p 5 (x-axis). (B) Proliferation response is shown as stimulation indices (y-axis) for Bet v 1 (x-axis). Pooled data from two independent experiments are shown. Results are presented as box plots and significant P values are indicated. (* p<0.05; ns, not significant). Note: data from the control group were also partially shown in (31). [file Image_6.tiff]
