# Supplementary material for: Adoptive transfer of allergen-expressing B cells prevents IgE-mediated allergy
Source: Front Immunol. 2023 Nov 23;14:1286638. doi: 10.3389/fimmu.2023.1286638 (PMC10703460; doi:10.3389/fimmu.2023.1286638)
Supplement: Supplementary Table 1 — Immunization and treatment protocol. Cell therapy, rapamycin, and antibody treatment were performed as shown in the table. Mice were sensitized with Phl p 5 and Bet v 1. Whole-body plethysmography (WBP) and cutaneous hypersensitivity testing (SPT) to Phl p 5 were performed at the given time points. [file Table_1.docx]

| **Group** | **n=** | **Cell therapy** | **Rapamycin and antibody treatment** | **Sensitization to Phl p 5 and Bet v 1** | ***In-vivo* endpoints** |
| --- | --- | --- | --- | --- | --- |
| Phl p 5^+^ B cells | 29 | 1 x 10^7^ isolated CD19^+^ B cells | Rapamycin (0.1 mg/mouse, d-1/0/2) + anti-CD40L mAB (1 mg/mouse, d0) | + | WBP w16 (n=13)  SPT w17 (n=7)  WBP w39 (n=6) |
| No cell transfer | 19 | - | - | + | WBP w16 (n=11)  SPT w17 (n=2)  WBP w39 (n=6) |
| No cell transfer + IS | 3 | - | Rapamycin (0.1 mg/mouse, d-1/0/2) + anti-CD40L mAB (1 mg/mouse, d2) | + | - |
| Phl p 5^+^ BM | 6 | 2 x 10^7^ unseparated BMC | Rapamycin (0.1 mg/mouse, d-1/0/2) + CTLA4Ig (0,5 mg/mouse, d2) | + | - |
| Phl p 5^+^ B +anti-CTLA4 | 5 | 1 x 10^7^ isolated CD19^+^ B cells | Rapamycin (0.1 mg/mouse, d-1/0/2) + anti-CD40L mAB (1 mg/mouse, d2) + anti-CTLA mAB (1 mg/mouse, d21, 0.5 mg, d23, 25 and 27) | + | SPT w17 (n=5) |
| Phl p 5^+^ B +anti-PD-1 | 4 | 1 x 10^7^ isolated CD19^+^ B cells | Rapamycin (0.1 mg/mouse, d-1/0/2) + anti-CD40L mAB (1 mg/mouse, d2) + anti-PD1 mAB (1 mg/mouse, d21, 0.5 mg, d23, 25 and 27) | + | SPT w17 (n=5) |
| Phl p 5^+^ CD4^+^ T cells | 3 | 1 x 10^7^ isolated CD4^+^ T cells | Rapamycin (0.1 mg/mouse, d-1/0/2) + anti-CD40L mAB (1 mg/mouse, d2) | + | - |
| Phl p 5^+^ CD8^+^ T cells | 3 | 1 x 10^7^ isolated CD8^+^ T cells | Rapamycin (0.1 mg/mouse, d-1/0/2) + anti-CD40L mAB (1 mg/mouse, d2) | + | - |
| Phl p 5^+^ T cells | 5 | 5 x 10^6^ isolated CD4^+^ T cells +  5 x 10^6^ isolated CD8^+^ T cells | Rapamycin (0.1 mg/mouse, d-1/0/2) + anti-CD40L mAB (1 mg/mouse, d2) | + | - |
| Phl p 5^+^ GM-CSF DC | 5 | 1 x 10^7^ GM-CSF stimulated DCs | Rapamycin (0.1 mg/mouse, d-1/0/2) + anti-CD40L mAB (1 mg/mouse, d2) | + | - |
| Phl p 5+ Ftlt3 DC | 6 | 1 x 10^7^ Flt3L stimulated DCs | Rapamycin (0.1 mg/mouse, d-1/0/2) + anti-CD40L mAB (1 mg/mouse, d2) | + | - |
| Phl p 5^+^ B +CTLA4Ig | 3 | 1 x 10^7^ isolated CD19^+^ B cells | Rapamycin (0.1 mg/mouse, d-1/0/2) + CTLA4Ig mAB (0.5 mg/mouse, d2) | + | - |
